# In Vivo Anti-Inflammatory and Antinociceptive Activities of Black Elder (*Sambucus nigra* L.) Fruit and Flower Extracts

**DOI:** 10.3390/ph17040409

**Published:** 2024-03-23

**Authors:** Daniela Seymenska, Desislava Teneva, Irina Nikolova, Niko Benbassat, Petko Denev

**Affiliations:** 1 Department of Pharmacognosy and Pharmaceutical Chemistry, Faculty of Pharmacy, Medical University-Plovdiv, 4002 Plovdiv, Bulgaria or dsdanymag@gmail.com (D.S.); niko.benbasat@mu-plovdiv.bg (N.B.); 2 Laboratory of Biologically Active Substances, Institute of Organic Chemistry with Centre of Phytochemistry, Bulgarian Academy of Sciences, 139 Ruski Blvd., 4000 Plovdiv, Bulgaria; desislava.teneva@orgchm.bas.bg; 3 Department of Pharmacology, Pharmacotherapy and Toxicology, Faculty of Pharmacy, Medical University-Sofia, 1000 Sofia, Bulgaria; inikolova@pharmfac.mu-sofia.bg

**Keywords:** black elder (*Sambucus nigra* L.), polyphenols, inflammation, anti-inflammatory activity, antinociceptive activity

## Abstract

*Sambucus nigra* L. (*S. nigra*, SN) or black elder is a traditional medicinal plant widely used worldwide for therapeutic and dietary purposes. The aim of the current study was to investigate the anti-inflammatory and antinociceptive activities of black elder fruit and flower extracts (SNFrE and SNFlE, respectively). The primary polyphenol constituents in the flower extract were flavonoids and phenolic acids, while anthocyanins were the main components in the fruit extract. SNFrE revealed pronounced and dose-dependent in vivo anti-inflammatory activity assessed by the cotton pellet-induced granuloma test. Doses of 10, 20, and 50 mg/kg BW of SNFrE reduced the weight of induced granuloma in rats by 20.3%, 20.5%, and 28.4%, respectively. At the highest dose (50 mg/kg BW), SNFrE had significant (*p* < 0.01) anti-inflammatory activity comparable to that of diclofenac, the reference compound used (10 mg/kg BW). In addition, the in vivo antinociceptive activity of the extracts in mice was estimated using the acetic-acid-induced writhing test. Both extracts at doses of 50 mg/kg BW inhibited the abdominal contractions induced by the acetic acid significantly comparing to the control group (*p* < 0.01). Our findings indicate that black elder extracts and particularly SNFrE possess anti-inflammatory and antinociceptive activities, providing experimental evidence for the use of *S. nigra* in traditional medicine.

## 1. Introduction

Inflammatory diseases have become a major factor in population morbidity. Often accompanied by pain [1,2], they are a significant problem that affects numerous people worldwide [3]. Chronic inflammation results in a long-term damage that leads to tissue and organ dysfunction [4,5]. Current therapy options for inflammatory diseases primarily include steroidal and nonsteroidal anti-inflammatory drugs (NSAIDs), as well as opioid analgesics for pain control. Corticosteroid use frequently results in hypertension, hyperglycemia, and osteoporosis [5,6]. Chronic NSAIDs use, on the other hand, has been associated with gastrointestinal issues, bleeding, and kidney damage [2,5,7]. Thus, it is necessary to seek for new anti-inflammatory and antinociceptive drugs with increased potency and fewer side effects. Botanical therapies containing natural products have long been of interest due to the potential synergistic therapeutic effects of the plant components [8]. Moreover, many diseases have diverse etiology, and a combination of herbal preparations could be a more effective treatment than a single drug administration [9]. Herbal preparations are often utilized in complementary and alternative medicine as anti-inflammatory agents [10,11,12]. People are now focusing on phytonutrients present in plants because of their ability to function as antioxidants or their anti-inflammatory (etc.) properties. The use of natural products as alternatives for inflammatory disease is considered safe, effective, biocompatible, and economical [13]. Plant polyphenols, including phenolic acids, flavonoids, and anthocyanins, have anti-inflammatory properties similar to NSAIDs and have gained academic attention as natural anti-inflammatory remedies with minimal adverse effects [14].

*S. nigra* L., commonly known as elderberry, black elder, or European elder, is a member of the Adoxaceae family (previously Caprifoliaceae). Its medicinal properties are recognized worldwide, and it is included in the pharmacopeias of several countries, including the European pharmacopeia [15]. Black elder is a deciduous shrub or tree between 4 and 12 m, which is widely distributed across Europe, western and central Asia, northern Africa, and North America [16,17,18]. It blooms between May and June with white-cream or greenish-yellow flowers, gathered in flat, complex, umbrella-like umbels [17]. Fruits are fleshy with a black-purple color and shiny skin. They usually ripen from July/August to October [18]. Flowers and fruits are the primary medicinal plant raw materials of black elder [19]. They contain a variety of bioactive substances, including polyphenols, flavonoids, and anthocyanins, which determine their health-promoting activities, such as antioxidant, anti-inflammatory, immunomodulatory, anticancer, antiviral, antimicrobial, etc. [16,20,21,22,23,24,25,26,27]. European elderberry extracts, juices, or syrups have become increasingly popular in the EU and US as remedies for cold and flu symptoms [26,28]. Currently, numerous health products containing European elder fruits, such as Sambucol, Sambucus for kids, and many others, are sold in drug and natural food stores. The use of these products is not fully supported by experimental or clinical data [28]. Therefore, it is important to reveal the biological properties of elderberries and elderflowers as highly valued raw materials.

Numerous components of elderberries and elderflowers have been determined; however, their biological activities have not been fully clarified. According to the European Medicines Agency (EMA) for elderberry preparations, complete information on their traditional use based on a specified posology is missing, as well as the necessary information on their specified strength [29]. Furthermore, various food supplements are derived from its fruits and flowers. Although some studies have reported in vitro anti-inflammatory activity of black elder [15,25,30], they were conducted primarily using in vitro cell models, and elderberry fruits and flowers have not been extensively studied for their anti-inflammatory effect in vivo in animal models. Apart from this, to the best of our knowledge, there are no in vivo studies on the antinociceptive activity of elderberry fruit and flower extracts. Therefore, the aim of the study was to investigate the anti-inflammatory and antinociceptive activities of *S. nigra* fruit and flower extracts. In a recent study, these extracts, and particularly the anthocyanin-rich black elder fruit extract, revealed pronounced activity against herpes simplex virus type-2 (HSV-2) [31]. The outcomes of our research, related to the anti-inflammatory and pain-relieving properties of the studied extracts, could have practical implications for the development of nutraceuticals with precise polyphenol composition and increased biological value.

## 2. Results and Discussion

### 2.1. Acute Toxicity of SNFrE and SNFlE

In the current study, the up-and-down method was used to determine the p.o. (peroral) and i.p. (intraperitoneal) lethal doses (LD_50_) of the extracts studied because it involves a smaller number of animals [32]. The dose range chosen was 125–5000 mg/kg BW. The oral LD_50_ of dry SNFrE was found to be 5000 mg/kg BW and the intraperitoneal LD_50_ was 500 mg/kg BW. The oral LD_50_ of dry SNFlE was 3000 mg/kg BW, and the intraperitoneal one was 300 mg/kg BW. Therefore, these doses can be classified as practically nontoxic in accordance with the Test Guidelines 425 (Up and Down Procedure) of the Organization for Economic Cooperation and Development (OECD) [32]. Thus, the doses of 10, 20, and 50 mg/kg BW were selected to be applied in the in vivo assays. Our results confirmed the safety assessment of *S. nigra* L. flowers and fruits, which have been recognized as a safe food additive by the US Food and Drug Administration (FDA) and the European Medicines Agency (EMA) [29,33]. Furthermore, Azevedo et al. have shown that anthocyanin-rich elderberry extract has a conducive toxicological profile and had no negative effects on the health of humans who consummated it and that it might even have some useful effects [34].

### 2.2. Anti-Inflammatory Activity of SNFrE and SNFlE

**Figure 1:**
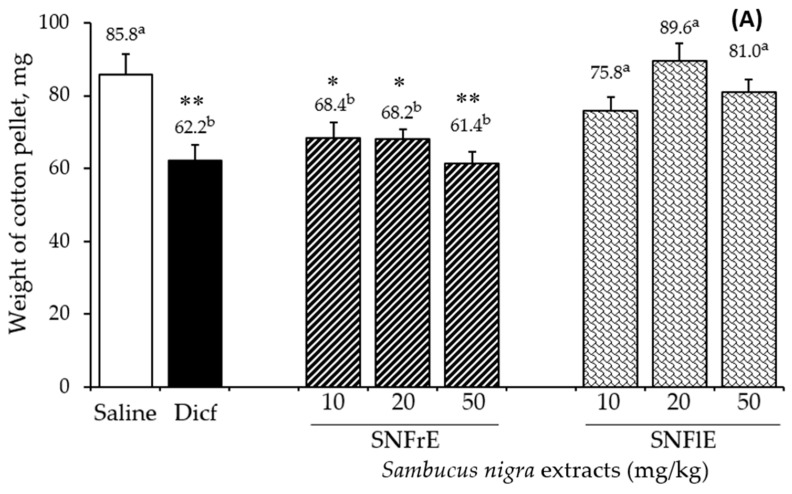

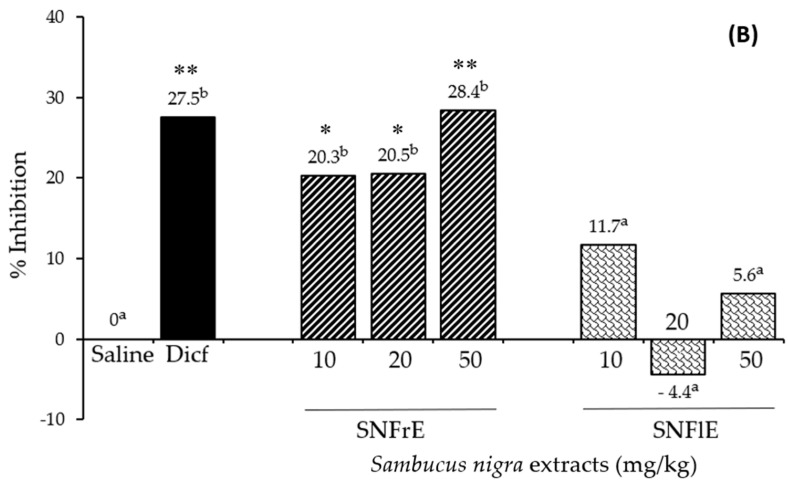
Anti-inflammatory activity of *Sambucus nigra* fruit and flower extracts (SNFrE and SNFlE, respectively) in the cotton-pellet-induced granuloma model. (**A**) Weight of cotton pellet. Values are expressed as mean granuloma weight ± S.E.M in mg, (*n* = 6). Statistical analysis was performed using one-way ANOVA followed by Tukey’s post hoc multiple comparison test. Mean values with different superscripted letters indicate significant differences (* *p* < 0.05, ** *p* < 0.01 vs. saline). (**B**) Percentage of inhibiting the mean cotton pellet weight in the different groups.

To support the claims of traditional medicine on the therapeutic benefits of *S. nigra* L., we investigated the anti-inflammatory activity of fruit and flower extracts using the cotton pellet granuloma test (picture of pellets is shown on Supplementary Figure S1). Oral administration of SNFrE (10, 20, and 50 mg/kg BW) produced a dose-dependent anti-inflammatory response, with significantly reduced granuloma weight. As shown in Figure 1A, the mean granuloma weight for doses of 10, 20, and 50 mg/kg BW of SNFrE were 68.4 ± 9.94, 68.2 ± 9.69, and 61.4 ± 10.22 mg, respectively, compared with the control group (saline). The weight of the cotton pellet-induced granuloma was reduced by 20.3%, 20.5%, and 28.4% at doses of 10, 20, and 50 mg/kg BW of SNFrE, respectively (Figure 1B). The reference drug (diclofenac sodium 10 mg/kg BW) produced significant anti-inflammatory activity with an inhibitory rate of 27.5%. The highest SNFrE dose (50 mg/kg BW) had significant (*p* < 0.01) anti-inflammatory activity, which clearly indicates that the extracts have the capacity to prevent development of granulomatous tissue. Interestingly, SNFlE in the three doses tested in the animal model used did not reveal an anti-inflammatory effect.

Granulomatous tissue reduction is a marker of the antiproliferative activity of NSAIDs. It is a common knowledge that diclofenac is widely used to treat chronic inflammatory diseases [35], inhibits cyclooxygenase (COX)-2 enzyme [36], and decreases the production of prostaglandins during the late stages of inflammation [37]. Suppression of the release of inflammatory mediators such as prostaglandins could probably be the mechanism underlying the anti-inflammatory effect of the extracts [38]. The arachidonic acid metabolic pathway, as well as the cytokine, NO, mitogen-activated protein kinase, and nuclear factor-kB (NF-κB) pathways are associated with the mechanism of anti-inflammatory and antinociceptive activities [39,40]. Different signaling pathways are mediated by cytokines (IL-1, IL-6, TNF-, NO, etc.) [41,42,43].

*Sambucus nigra* fruit and flower extracts used in the current study were particularly rich in phenolic compounds, as reveled by our recent study [31]. The total polyphenol content of the fruit and flower extracts was 59,596.6 ± 1796.3 mg/100 g DW and 42,647.1 ± 1331.8 mg/100 g DW for SNFrE and SNFlE, respectively. The HPLC analysis revealed the presence of several classes of phenolic compounds: hydroxycinnamic acids, hydroxybenzoic acids, flavonols, and anthocyanins. Chlorogenic acid was the main phenolic acid found in SNFlE and SNFrE, with 7086.7 ± 312.8 mg/100 g DW and 3614.9 ± 190.1 mg/100 g DW, respectively. The quantity of neochlorogenic acid in the flower extract (1342.2 ± 69.2 mg/100 g DW) was three times higher than in the fruit extract (417.7 ± 16.3 mg/100 g DW). Compared to other phenolic acids, the amount of benzoic acid in SNFrE was also relatively higher (1038.2 ± 92.6 mg/100 g DW). Flavonols (quercetin-3-rutinoside and quercetin-3-glucoside) were the predominant group of flavnoids in both extracts, followed by the aglicones myricetin and quercetin. Rutin was the flavonol with the highest total mean content: 14,232.1 ± 648.9 and 4623.0 ± 283.7 mg/100 g DW in SNFlE and SNFrE, respectively. Notably, the polyphenol content of the fruit extract was found to be about 1.4 times higher than that of the flower extract, due to the high content of anthocyanins: 50,557.1 mg/100 g DW, of which there are 24,341.1 ± 1017.4 mg/100 g DW cyanidin-3-glucoside and 21,051.4 ± 951.5 mg/100 g DW cyanidin-3-O-sambubioside [31]. Many medicinal plants, rich in polyphenols, have shown anti-inflammatory activity in pharmacological experiments [44], and polyphenols have shown an ability to suppress prostaglandin pathways [13,37,45]. For example, flavones effectively reduce the synthesis of cyclooxygenase-2 [46,47], whereas flavonoids can inhibit eicosanoids such as prostaglandins, decreasing inflammation and pain in cancer patients [48]. Several studies have demonstrated black elder’s anti-inflammatory effects exhibited via the decreased production of pro-inflammatory cytokines, including IL-6 and TNF-α, and reduced neutrophil activation [44,49,50]. Their results indicate that European elder can reduce local inflammation, suppress the release of pro-inflammatory cytokines, inhibit the production of ROS [20], and suppress the NF-κB [49]. Unlike the fruit extract, which revealed a pronounced dose-dependent effect, the flower extract did not show anti-inflammatory activity at any of the three concentrations tested. This cannot be attributed solely to the higher phenolic content of SNFrE since it is known that elderberry polyphenols differ in their activity. For example, a recent study used molecular docking to demonstrate that the anti-inflammatory effect of elderberry polyphenols was due mainly to caffeic and homovanillic acids [51]. The most obvious difference in the chemical composition of the used extracts was the significant amount of anthocyanins in SNFrE [31], which indicates the role of these compounds in the observed effect. It is known that anthocyanins can exert anti-inflammatory effects via several mechanisms. For example, these phenolic compounds reduce inflammation via the inhibition of COX-2 expression in lipopolysaccharide (LPS)-activated RAW 264 cells [52,53]. Seeram et al. have reported the inhibition of cyclooxygenase by the cyanidin glycosides present in different berries [54]. Other sources have confirmed the involvement of anthocyanins in the production of pro-inflammatory components (e.g., transcription factors, NF-κB, pro-inflammatory cytokines) and the modulation of pro-inflammatory gene expression [52]. Some of these effects are likely to be due to the antioxidant properties of anthocyanins and the neutralization of ROS. Therefore, Wang et al. and Ma et al. supposed that in addition to the signaling pathway involved in ROS scavenging, other signaling pathways were also involved in the anti-inflammatory effect induced by anthocyanins [53,54,55].

Our results demonstrate in vivo the dose-dependent prevention of subacute inflammation by polyphenol-enriched black elder fruit extract (but not by flower extract), most probably by interfering with the metabolism of arachidonic acid. The results are in line with the larger number of reports in the literature and imply that the anti-inflammatory effect of SNFrE may include a number of diverse mechanisms, including inhibition of the synthesis of inflammatory mediators and/or their release via COX or other particular enzymatic mechanisms. However, further studies involving inflammatory mediators and COX inhibition should be conducted.

### 2.3. Antinociceptive Activity of SNFrE and SNFlE

**Figure 2:**
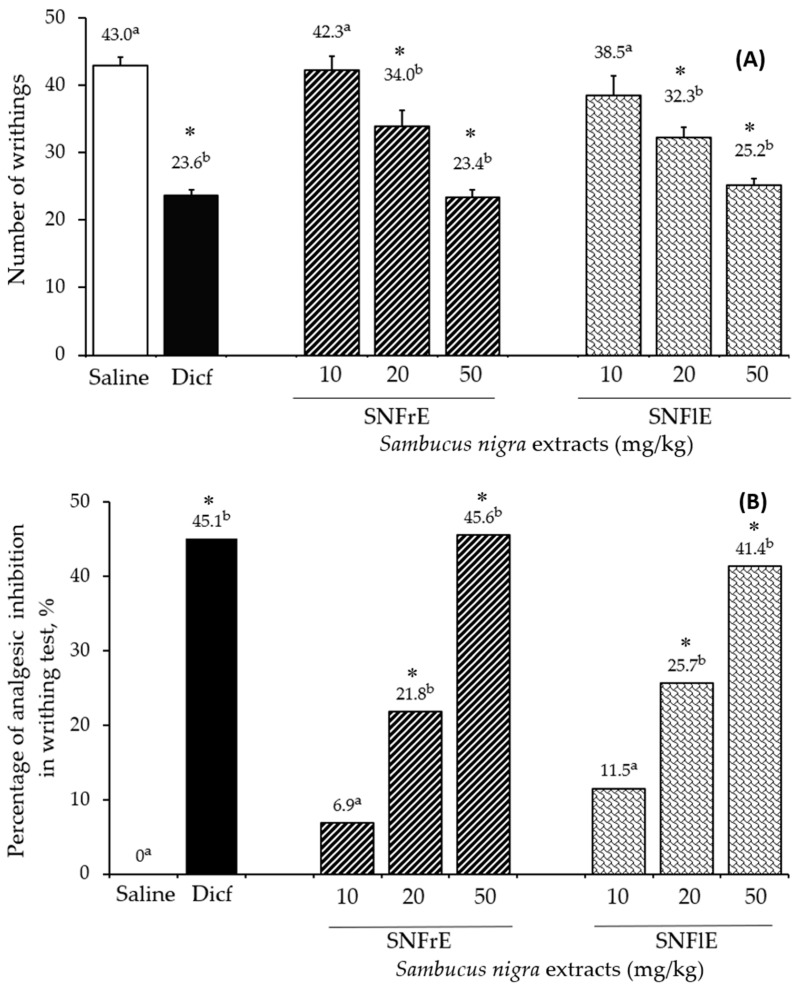
Antinociceptive activity of *Sambucus nigra* fruit and flower extracts (SNFrE and SNFlE, respectively) in acetic-acid-induced abdominal writhing in mice. (**A**) Number of writhes: values are expressed as mean ± S.E.M. (*n* = 6). Statistical analysis was performed using one-way ANOVA followed by Tukey’s multiple comparison test. Mean values with different superscripted letters indicate significant differences (* *p* < 0.01 vs. saline). (**B**) Percentage of inhibition in the number of acetic-acid-induced abdominal writhes in mice from the different groups.

The writhing test was used to assess the analgesic effect of black elder fruit and flower extracts and compare it to diclofenac, a widely used analgesic and anti-inflammatory drug. SNFrE and SNFlE demonstrated a dose-dependent analgesic effect in the writhing test (Figure 2). Acetic acid is commonly used to evaluate visceral pain because it causes abdominal contraction and releases endogenous pain mediators [3]. Oral administration of SNFrE and SNFlE (10, 20, and 50 mg/kg BW) produced a dose-dependent antinociceptive response, with significantly (*p* < 0.01) reduced writhing induced by acetic acid. As shown in Figure 2A, the reference drug (diclofenac sodium 10 mg/kg) reduced the number of writhes to 23.6 ± 0.85, whereas SNFrE and SNFlE reduced it to 23.4 ± 1.14 and 25.2 ± 0.95, respectively (*p* < 0.01, as compared to the control group). Furthermore, the reference drug produced significant (*p* < 0.01) antinociceptive activity with an inhibitory rate of 45.1% (Figure 2B). SNFrE and SNFlE decreased the acetic-acid-induced abdominal pain by 45.6% and 41.4%, respectively, at the highest dose administered (50 mg/kg BW). Treatment with SNFrE and SNFlE at a dose of 20 mg/kg BW also significantly inhibited the number of writhes (34.0 ± 2.31 and 32.3 ± 1.52, respectively) and decreased the acetic-acid-induced abdominal pain (by 21.8% and 25.7%, respectively), as compared to saline (*p* < 0.01).

According to Młynarczyk et al., elderberries exhibit a mild analgesic effect and can be employed as an adjuvant pain reliever for neuralgic, migraine, and sciatica pain [26]. In our study, European elder antinociceptive activity was analyzed in a model allowing for the evaluation of responses to chemically induced pain [38,56]. Although acetic-acid-induced abdominal pain is not a typical model, it has been extensively utilized for screening analgesic drugs due to its resemblance to the symptoms of human visceral pain. The acetic-acid-induced writhing test is commonly used to investigate the way in which medications (opioids, non-steroidal anti-inflammatory medicines, antispasmodics, antihistamines, calcium channel blockers) [57] and plant extracts [3,58] affect peripheral antinociception. It has been proposed that intraperitoneal acetic acid injection induces the secretion of endogenous mediators such as prostaglandins, including prostaglandin E2, in peritoneal tissues. Prostaglandins induce abdominal constriction, forelimb extension, and body lengthening by activating and sensitizing peripheral chemosensitive nociceptive receptors and are known to be related to inflammatory pain [3,59]. These activities play an important role in the progress of peripheral inflammation [38]. Acetic acid induces nociception arising in the resident cells (macrophages and mast cells) present in the peritoneal cavity. These cells produce cytokines (TNF-α, IL-1, and IL-8) that assist the straining response. This model, however, is unable to determine whether the black elder antinociceptive effect is peripheral or central [60,61]. As far as we know, our study is the first one to investigate, in vivo, the antinociceptive activities of *S. nigra* extracts, thus expanding experimental data into the biological effects of black elder fruits and flowers.

### 2.4. Histopathology

**Figure 3:**
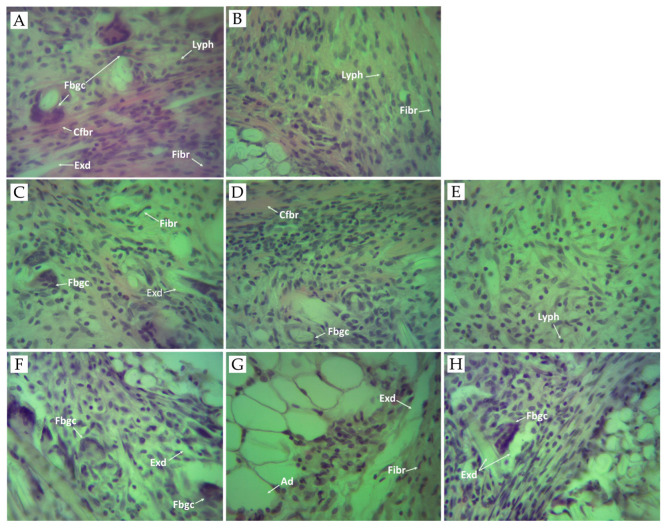
Photomicrographs of granulomatous tissue. (**A**) Control group received sterile 0.9% saline. Histological section of granulomatous tissue showing acute inflammation with necrosis, exudates (Exd), foreign body giant cells (Fbgc), collagen fiber (Cfbr), fibroblasts (Fibr), and lymphocytes (Lyph). (**B**) Positive control group treated with diclofenac (10 mg/kg BW). Histological section of granuloma tissue showing mild chronic inflammation with fibroblasts (Fibr), and lymphocytes (Lyph). (**C**) Experimental group treated with SNFrE 10 mg/kg BW. Histological section of granuloma tissue showing chronic inflammation with a single foreign body giant cell (Fbgc), exudates (Exd), and fibroblasts (Fibr). (**D**) Experimental group treated with SNFrE 20 mg/kg. (**E**) Experimental group treated with SNFrE 50 mg/kg BW. The histological section of tissue does not show foreign body giant cells (Fbgc). (**F**) Experimental group treated with SNFlE 10 mg/kg BW. Histological section of granuloma tissue showing chronic inflammation with foreign body giant cells (Fbgc) and exudates (Exd). (**G**) Experimental group treated with SNFlE 20 mg/kg BW. Histological section of granuloma tissue showing chronic inflammation with exudates (Exd), fibroblasts (Fibr), and adipocytes (Ad). (**H**) Experimental group treated with SNFlE 50 mg/kg BW. Histological section of granuloma tissue showing moderate inflammation with exudates (Exd) and a single foreign-body-type giant cell (Fbgc). Sections were stained with H&E (hematoxylin and eosin); magnification ×40.

Granulomatous inflammation is a type of chronic inflammation that is distinguished by granulomas, which are microscopic aggregates of macrophages turned into epithelioid cells and surrounded by fibroblasts, lymphocytes, and plasma cells [62]. Chronic inflammation results from the continuous effects of inflammatory factors and cotton pellet-induced granuloma in test animals is a common method used to determine and study chronic inflammation and its effect on macrophage function [63,64]. The histopathologic findings regarding the inflammatory process (Figure 3) were examined after the rats were sacrificed. In our study, granuloma tissue was formed in all tested animals due to the implantation of subcutaneous cotton pellets. The macroscopic analysis of the biopsy materials obtained revealed the presence of gray-white tissue surrounding the cotton implants. It was comprised of macrophages, epithelioid cells, and foreign-body-type giant cells. The presence of granulomatous tissue and macrophages are signs of a chronic inflammatory reaction [64].

In the negative control group (Figure 3A), the granulomatous tissue showed acute inflammation with necrosis and exudates, giant cells, and lymphocytes. Fibrous tissues comprising lymphocytes and plasma cells were observed. As compared to the control group, the morphological structure of the foreign-body-type granuloma in the 10 mg/kg-diclofenac-treated rats (Figure 3B) showed mild chronic inflammation with a noticeable decrease in the area of granulomatous and inflammatory exudates, without the presence of foreign-body-type giant cells. Fibroblasts were observed in the periphery of the granuloma. Decreasing granuloma tissue and preventing the occurrence of the collagen fiber are indicators of the antiproliferative effect of NSAIDs (incl. diclofenac) [65]. The presence of chronic inflammatory reaction was evident in the 20 mg/kg-flower-extract-treated group (Figure 3G). The histological section of the granulomatous tissue in the 50 mg/kg-flower-extract-treated group (Figure 3H) showed moderate inflammation with exudates and a single foreign body-type giant cell. Interestingly, animals that received SNFrE at a dose of 50 mg/kg also showed significant (*p* < 0.01) reduction in the size of the granulomatous lesions, as shown by the decrease in the amount of inflammatory exudates and the increase in the number of collagen fibers. Thus, the decrease in granuloma weight indicates suppression of the proliferative phase of inflammation, which was the most effectively inhibited by SNFrE et a dose of 50 mg/kg BW.

**Figure 4:**
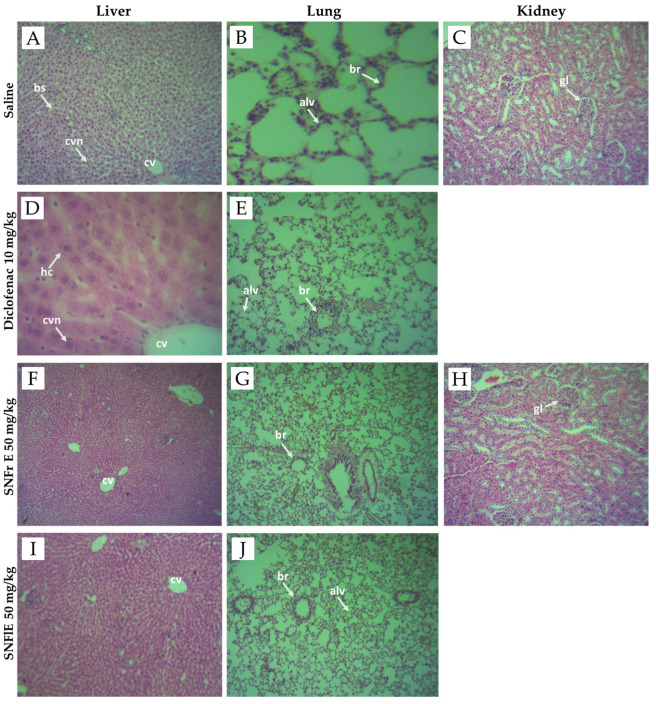
Photomicrographs of liver, lung, and kidney tissues from negative controls (images **A**–**C**), diclofenac-treated group (images **D**,**E**), groups treated with *S. nigra* fruit extract 50 mg/kg BW (images **F**–**H**) and flower extract 50 mg/kg BW (images **I**,**J**). Images (**A**,**D**,**F**,**I**) of histological structure of liver showing normal liver architecture including central vein (cv), central vesicular nuclei (cvn), blood sinusoids (bs), and hepatic cells (hc). Images (**B**,**E**,**G**,**J**) of normal histological architecture of lung showing normal intrapulmonary bronchioles (br) and alveoles (alv). Images (**C**,**H**) of histological structure of kidney showing normal mice kidney architecture, showing normal glomerulus (gl). Sections were stained with H&E (hematoxylin and eosin), magnification ×40.

In our study, no differences were found in the morphological structure of the lung, kidney, and liver biopsy samples obtained from the control and experimental groups (Figure 4). Figure 4 shows that all of the above organs have well-organized cellular structures without obvious abnormality. The liver sections from the control, diclofenac-treated, SNFrE-, and SNFlE-treated groups showed normal liver architecture with normal hepatocyte morphology and no evidence of pathological damage (Figure 4A,D,F,I). The lung tissue photomicrographs of the control, diclofenac-treated, and extract-treated groups showed normal histological architecture, normal intrapulmonary bronchioles, and no pathological changes (Figure 4B,E,G,J). The kidney histological analysis of the 50 mg/kg-SNFrE-treated group (Figure 4H) showed no change in the histological architecture of the glomerular and renal tubules as compared to the control group (Figure 4C).

In conclusion, our results of the histopathological examinations confirmed the safety assessment of FDA and EMA for *S. nigra* L. flowers and fruits.

## 3. Materials and Methods

### 3.1. Chemicals

All chemicals used in the present study were of analytical and pharmaceutical grade. Diclofenac sodium, a widely used NSAID, was used as a reference and was delivered by Sigma-Aldrich (Steinheim, Germany). All other reagents and solvents used were purchased from local distributors.

### 3.2. Plant Materials and Extracts Preparation

*S. nigra* (L.) flowers and fruits were collected from natural populations on the territory of the Rodopi mountain (Ravnogor village), Bulgaria, in June 2019. The taxonomic identification of *Sambucus nigra* L. species was performed according to the respective monograph in the European Pharmacopoeia [66] based on diagnostic macroscopic features, with the assistance of the Department of Pharmacognosy and Pharmaceutical Chemistry, Faculty of Pharmacy, Medical University—Plovdiv (assoc. prof. Niko Benbassat, PhD). The procedure for preparation and purification of SNFrE and SNFlE and their phytochemical composition are described in detail in our previous research [31]. Briefly, both *S. nigra* flowers and fruits were extracted with 60% (*v*/*v*) ethanol. The extracts were filtered and concentrated by vacuum evaporation and ethanol-free liquids underwent purification by solid-phase extraction as described by Denev et al. [67]. Extracts were frozen, lyophilized, and stored at −18 °C [31].

### 3.3. In Vivo Experiments

#### 3.3.1. Ethical Statement

All experiments were performed in full accordance with the respective Bulgarian and European Guidelines for the Care and Use of Laboratory Animals. Permission to use animals in the experiment was obtained from the Food Safety Agency at the Bulgarian Ministry of Agriculture and Food. The protocol (No. 212/2018) for the study was approved by the Ethical Committee on Animal Experimentation of the Medical University, Sofia.

#### 3.3.2. Experimental Animals

Forty-eight Wistar male rats, strain H, weighing approximately 200–250 g, and a total of eighty-eight Swiss Albino mice (18–22 g) purchased from Slivnitza Animal House (Bulgaria) were used in the in vivo experiments. The animals were housed in an animal house facility under standard laboratory conditions (water and food *ad libitum*; 12 h dark/light cycle; 22 ± 3 °C room temperature and 50% humidity). Before treatment, they were allowed to acclimatize to the laboratory conditions for 7 days. The experimental animals were randomly divided into eight groups, each consisting of six animals (*n* = 6). The animals were treated with saline, diclofenac, and extracts p.o. as follows:

Group 1: negative control group of animals receiving only saline.

Group 2: positive control group of animals, treated with diclofenac at a dose of 10 mg/kg BW.

Groups 3, 4 and 5: animals treated with elderberry fruit extract at a dose of 10, 20, and 50 mg/kg BW, respectively.

Groups 6, 7 and 8: animals treated with elderberry flower extract at a dose of 10, 20 and 50 mg/kg BW, respectively.

#### 3.3.3. Acute Toxicity and Index of Absorption

Acute p.o. and i.p. toxicity, as well as LD_50_, were estimated in the experimental mice in accordance with Test Guidelines 425 (Up and Down Procedure) of the Organization for Economic Cooperation and Development (OECD) [32]. The LD_50_ assay was used to investigate acute toxicity. This test involves administering compounds to mice in amounts that grow in a geometric progression to find the dose at which 50% of the mice in the experiment would die. Forty Swiss Albino mice were used. The animals were administered with polyphenol-enriched fruit and flower extracts of *S. nigra* (125–5000 mg/kg BW). Each animal was treated once daily orally or by i.p. injection and was observed for clinical signs or mortality. At the end of the 14-day observation period, all mice were euthanized under ether anesthesia and all organs and tissues were macroscopically examined. The index of absorption (IA) was calculated as a ratio of LD_50_ i.p./LD_50_ p.o.

#### 3.3.4. Cotton Pellet-Induced Granuloma

The in vivo anti-inflammatory activity of black elder fruit and flower dry extracts was evaluated by the cotton pellet granuloma test in rats, as described by Koti et al. and Ismail et al. [68,69]. The granulomatous lesions were induced by surgically implanting a cotton pellet subcutaneously in the dorsal region of the rats. Sterile pellets of cotton, weighing 20 ± 1 mg each, were aseptically implanted in the interscapular distance under the skin on the back of the rats following anesthesia. The animals of all groups were treated once daily throughout the 7-day experimental period. The rats were euthanized on the eighth day after implantation, and the pellets surrounded by granulomatous tissue were dissected carefully out and dried. The mean weight of the granulomatous tissue formed around each pellet was recorded. The pellets were weighed both moist and dry. The weight of the pellets obtained from the animals of the negative control, positive control, and black elder fruit and flower extract-treated groups were compared. The percentage in the change in granuloma weight relative to the control groups was calculated. The granulomas were examined under a microscope.

#### 3.3.5. Acetic-Acid-Induced Writhing Test

The analgesic property of the extracts was studied by using the acetic-acid-induced writhing test in mice. It was performed as originally described by Siegmund et al. [70]. The writhing test was performed 5 days after administration of the extracts by an intraperitoneal (i.p.) injection of 0.3% acetic acid at a dose of 0.1 mL/10 g BW. The number of writhes (including abdominal contractions, trunk twisting, and extension and elongation of the body and limbs) was determined from 5 to 15 min following the acetic acid injection. The analgesic activity was expressed as a percentage (%) of writhing inhibition and was determined using the formula given below:

Writhing inhibition = [(*Wc* − *Wt*)/*Wc*] × 100,

where *Wc* is the mean number of writhes of the negative control group and *Wt* is the mean number of writhes of the test sample.

#### 3.3.6. Histological Analysis

At the end of the investigation, the animals were euthanized, the granuloma tissue samples were collected for histological investigation, and sections of liver, lung, and kidney tissues were taken from definite groups in the study. The samples were fixed in 4% buffered paraformaldehyde, followed by embedding in paraffin, and the sections were stained with hematoxylin and eosin (H&E) [71,72]. All samples were then examined under a microscope (Leica DM 5000B, Wetzlar, Germany).

### 3.4. Statistical Analysis

The in vitro results were obtained via two independent experiments performed in duplicates and triplicates. The statistical analysis of data was performed using MS Excel 2016 software and expressed as mean ± SD. The in vivo data were presented as mean ± standard error of mean (S.E.M). The significant differences between the test and control groups were assessed via one-way analysis of variance with multiple comparisons (ANOVA), followed by Tukey’s post hoc test. The *p*-values lower than 0.05 and 0.01 (*p* < 0.05 and *p* < 0.01) were considered as statistically significant.

## 4. Conclusions

The current study demonstrates for the first time that *Sambucus nigra* fruit extract exhibited a dose-dependent anti-inflammatory effect in the in vivo cotton-pellet-induced granuloma test, which was confirmed via histopathological examination. Interestingly, the flower extract did not show anti-inflammatory activity. Both extracts revealed a potent dose-dependent analgesic effect, which was comparable to the effect of the analgesic and anti-inflammatory drug diclofenac at the highest dose tested (50 mg/kg BW). However, the mechanisms underlying these effects were not investigated in our study and remain unclear. The lack of toxicity indicates that elderberry fruit extract is suitable for long-term use as a dietary supplement in adjuvant therapy and is a promising candidate for the treatment of pain and inflammation. Further research is needed in order to identify the pure component(s) that cause the observed biological effects and to characterize its toxicity profile in longer-term use.

## Data Availability

Data are contained within the article.

## Supplementary Materials

The following supporting information can be downloaded at https://www.mdpi.com/article/10.3390/ph17040409/s1: Figure S1: Photograph of surgically removed cotton pellets after treatment with saline (C), diclofenac (D), fruit (Fr), and flower (Fl) extracts of *S. nigra*.
