# Supplementary material for: In Vivo Anti-Inflammatory and Antinociceptive Activities of Black Elder (Sambucus nigra L.) Fruit and Flower Extracts
Source: Pharmaceuticals (Basel). 2024 Mar 23;17(4):409. doi: 10.3390/ph17040409 (PMC11054073; doi:10.3390/ph17040409)

Supplementary materials

In Vivo Anti-Inflammatory and Antinociceptive Activities of Black Elder (*Sambucus nigra* L.) Fruit and Flower Extracts

Daniela Seymenska, Desislava Teneva, Irina Nikolova, Niko Benbassat and Petko Denev

Figure S1. Photograph of surgically removed cotton pellets after treatment with saline (C), diclofenac (D), fruit (Fr) and flower (Fl) extracts of *S. nigra.*

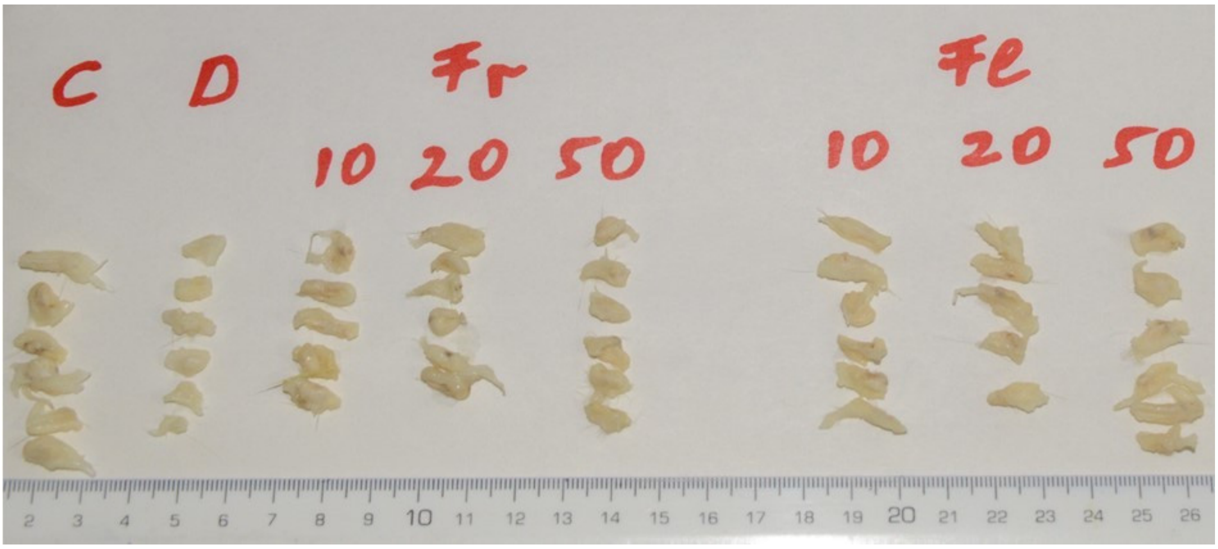

Supplement: Supplementary file 1 [file pharmaceuticals-17-00409-s001.zip › pharmaceuticals-2915971-supplementary.pdf]
